# Reversal Effect of Dihydromyricetin on Multiple Drug Resistance in SGC7901/5-FU Cells

**DOI:** 10.31557/APJCP.2020.21.5.1269

**Published:** 2020-05

**Authors:** Mingcai Wu, Ming Jiang, Ting Dong, Lei Xu, Jun Lv, Mengya Xue, Mengzhu Huang

**Affiliations:** 1 *Department of Biochemistry, Wannan Medical College, Wuhu, Anhui, P.R.China. *; 2 *Anhui Province Key Laboratory of Active Biological Macromolecules, Wuhu, Anhui, P.R.China. *; 3 *Wuhu second Sanatorium for Retired Cadres, Anhui military area, Wuhu, Anhui, P.R. China. *; 4 *Encephalopathy Center, The First Affiliated Hospital of Anhui University of Chinese Medicine, Hefei, Anhui, P.R. China. *

**Keywords:** Drug resistance, multiple, apoptosis, stomach neoplasms

## Abstract

**Background:**

One of the most common treatment for gastric cancer is chemotherapy, however, multiple drug resistance (MDR) induce the therapeutic effect which result in the failure of anticancer therapy. Dihydromyricetin (DMY) was reported to have antitumor activities on various human cancer cells in vitro, our previous studies demonstrated that DMY combined with mitomycin has inhibitory effect on proliferation of gastric carcinoma cells. However, the underlying role of DMY reversing the MDR of gastric carcinoma is poor understood. The aim of this study was to evaluate the reversal effect of DMY on MDR and investigate the molecular mechanisms in vitro.

**Methods:**

Using MTT assay, we identified the toxicity of DMY on SGC7901 and SGC7901/5-FU cells. The effect of DMY on 5-FU induced apoptosis was evaluated by flow cytometry analysis. Using RT-PCR and Western blot, we determined the MDR1 mRNA and protein expression.

**Results:**

DMY induced growth inhibition in both SGC7901 and SGC7901/5-FU cells, the IC50 value was 13.64±1.15 µg/mL, 20.69±1.82 µg/mL respectively. DMY treatment sensitized SGC7901/5-FU cells to cytotoxicity of 5-FU. The combination of DMY with 5-FU increased the apoptosis rate (9.91%, 16.67%) comparing with 5-FU alone (5.25%). Comparing with the control group, the MDR1 mRNA and protein expression in SGC7901/5-FU cells after treatment of DMY decreased significantly (P< 0.05).

**Conclusion:**

In brief, our study demonstrated that DMY effectively reversed multi-drug resistance occurring in SGC7901/5-FU cells cultured in vitro, and the potential mechanism was involved in the downregulation of the *MDR1* expression.

## Introduction

Stomach cancer is the fourth most common cancer worldwide with 930,000 cases diagnosed in 2002 (Parkin et al., 2002). It is a disease with a high death rate (~800,000 per year) making it the second most frequent leading cause of cancer death worldwide after lung carcinoma. It is more common in men and in developing countries (Torre et al., 2012; Ferlay et al., 2012). 

At present, an important therapeutic option for gastric cancer patients is chemotherapy. However, multidrug resistance (MDR) causes the failure of chemotherapy. MDR occurred in cancer cells which are exposed to an anticancer drug and acquired resistance to other multiple anticancer drugs with unrelated structure and mechanism, comparing with the drug-sensitive cancer cells, the intracellular drug concentration in the MDR cancer cells accumulated significantly lowly (Gottesman et al., 2002; Wu et al., 2018; Supawat et al., 2019). Searching for effective drug against clinical MDR has been a new promising strategy. However, patients treated with some MDR reversal agents may suffer from the unacceptable side effects. Recently, natural products has been applicated in the prevention and therapy of gastric cancer (Xu et al., 2018; Park et al., 2018). Ampelopsis grossedentata is a wild, flavonoid–rich plant found in Southern China, Its leaves can be used as food and medicinal materials. Dihydromyricetin (DMY), a 2,3-dihydroflavonol compound, is extracted from Ampelopsis grossedentata. A number of pharmacological studies have shown that tea vine is capable of regulating blood sugar and blood fat, anti-inflammatory, anti-oxidation, anti-virus, anti-tumor effects, etc (Chen et al., 2015; Hayashi et al., 2005; Murakami et al., 2004; Fan et al., 2018), and the anti-tumor effect has become one of the research hotspots in recent years. In the previous studies, we revealed that DMY combined with mitomycin induced apoptosis and the growth inhibition of gastric carcinoma SGC7901 cells compared to using mitomycin alone (Wu et al., 2011). However, no study has reported on the reversal effect of DMY on multidrug resistance in gastria carcinoma. Thus, the aim of the present study was to investigate the potential effect of DMY reversing drug resisitance in gastric cancer chemotherapy, and explore its underlying mechanisms utilizing multi-drug resistant human gastria carcinoma SGC7901/5-FU cells .

## Materials and Methods

*Cell culture*

Human gastric adenocarcinoma cell line SGC7901 and drug-resistance gastric cancinoma cell line (SGC-7901/5-FU) were procured from the Cell Bank of Chinese Academy of Sciences (Shanghai, China). All cells were cultured in RPMI-1640 medium supplemented with 10% fetal calf serum in a humidified incubator supplied with 5% CO_2_ at 37^o^C. 10 μg/ml 5-fluorouracil was added to the SGC7901/5-FU cells medium for obtaining the resistance phenotype, before experimentation performed, cells was grown in drug-free above-mentioned medium for at least 2 days. 

*Intrinsic cytotoxicity assay*

The cytotoxicity of DMY was analyzed using the 3-(4,5-dimethyl-2-thiazolyl)-2,5-diphenyl-2H-tetrazolium bromide (MTT) assay. Briefly, 1× 10^4^ cells growing at exponential phase were inoculated into each 96-well cell culture plate. The culture medium was removed after 24h culture at 37°C, 5% CO_2_. DMY was added and the final concentrations were 0, 0.625, 1.25, 2.5, 5, 10, 20 and 40 ug/mL. Then cells were exposed to 5% CO_2_ at 37°C for an additional 48h. Each well was then added with 20 μL MTT solution (5 mg/mL) and grown in a incubator in the dark for another 4h. The crystals were dissolved by 150 μL of DMSO (Sigma, USA) after discarding the supernatants. The absorbance (A) of each well was measured at an ELISA plate reader (Bio-Rad, USA) setting a wavelength of 490nm. The inhibition of cell grown was calculated by the following formula: Relative cell inhibitory rate (%) = [1–(mean A490 of experimental cells)/(mean A490 of control cells) ]×100%.

*Resisitance assay*

To assess the MDR of SGC7901/5-FU cells to chemotheraphy drugs, the parent SGC7901 cells and drug-resistant SGC7901/5-FU cells growing at log phase were inoculated into 96-well plates and cultured in various concentrations of 5-fluorouracil diluted in the RPMI1640 medium for 48h. The cytotoxicity of 5-fluorouracil was measured by MTT assay. Then got the 50% growth inhibition concentration (IC50) and Resistance index (RI). RI was used as an important metric to evaluate the resistant effect of MDR cells to various anticarcinogen. RI was calculated with the following formula: RI = the IC50 for the MDR cells/the IC50 for the parental cells.

*Reversal effect assays*

To evaluated the reversal effect to drug resistance of DMY in SGC7901/5-FU cells, the SGC7901/5-FU cells (1×10^4^ cells/well) were incubated in 96-well plates, and then treated with DMY, various concentrations of 5-fluorouracil, or combinations of DMY with 5-fluorouracil 24 h later. DMY was used at concentrations with low cytotoxicity. After the cells were incubated for 48h, MTT assay was performed. The reversal fold (RF) (Jin et al., 2005) were analyzed by the following formulas: RF= IC50 anticancer drug alone/ IC50 (anticancer drug + modulator) 

*Semi-quantitative RT-PCR*

According to the manufacturer’s instructions, total cells RNA was isolated with TRIzol reagent (Invitrogen, USA). The RNA pellet obtained in the final step was dissolved in 50 μL of sterile diethylpyrocarbonate (DEPC)-treated water, and its concentration was determined using a UV spectrophotometer at 260 nm. RNA was kept in DEPC-treated water at -70^o^C until use. Total RNA(1 μg) from each sample was reverse transcribed by the Access RT-PCR System (Promega, USA). The PCR primers sequences of target genes used were as follows: MDR1 forward:5’-AGACATGACCAGGTATGCCTAT-3’ and reverse 5’-AGCCTATCTCCTGTCGCATTA-3’; β-actin forward: 5’-CGCGAGAAGATACCCAGAT-3’ and reverse 5’-GCACTGTGTTGGCGTACAGG-3’. The refrence gene β-actin mRNA level was determined as a loading control. 1.5% agarose gel supplemented with 5 μg/mL of ethidium bromide was used to visualize the PCR products. The results were analyzed by gel imaging system. The relative mRNA expression of target gene was calculated by the following formula: The gray value of target gene/The gray value of housekeeping gene.

*Apoptosis assay*

After DMY treatment for 48 h, the cells were harvested and suspended by trypsin. Cell pellets were rinsed twice with cold PBS by centrifuging for 5 min at 1,000 rpm. Cells were resuspended in 500 μL of an Annexin V binding buffer (BioVision, Annexin V-FITC Reagent Kit) containing 5 μL of Annexin V-fluorescein isothiocyanate (FITC) and 5 μL of propidium iodide (PI). After incubation for 5 min at room temperature free of the light, the samples were analyzed using a FACS Calibur flow cytometer (BD, USA).

*Western blotting analysis*

Protein expression was determined by Western blotting. Cells in treatment groups and control group were havested and washed with cold PBS, solubilization buffer was used to generate the cell lysates, then collected the supernatant fraction by centrifuging for 15 min at 4^o^C. The bicinchoninic acid (BCA) protein assay was performed to estimate the protein concentration. In brief, 50 μg protein of each sample was separated by SDS-PAGE, and the gels were electrotransferred to PVDF membranes (Millipore, Bedford, MA, USA). The membrane was blocked for 1 h with PBST (0.05% Tween 20 dissolved in PBS) containing 5% non-fat milk, and then incubated with rabbit monoclonal antibody to P-gp and β-actin (1:500 and 1:1,000 dilution respectively, Santa Cruz Biotechnology, Inc, USA) for overnight at 4^o^C or 1 h at room temperature. Next, the membranes were washed with TBST three times and then incubated at room temperature for 1 h with a horseradish peroxidase conjugated anti-rabbit secondary antibody (1:1,000, Santa Cruz Biotechnology, Inc, USA). After washing the memberane three times, enhanced chemiluminescence method was performed to develop the immunoreactive bands, using the expression level of β-actin as a loading control.

*Statistical analysis*

SPSS 17.0 software were performed to statistical analysis, All quantitative data were obtained from 3 or more independent experiments and were depicted as means ± SD. Student’s t test or ANOVA was used to detect statistical comparisons between groups. Values of P less than 0.05 were considered to adjust a significant difference.

## Results

*DMY induced growth inhibition in both SGC7901 and SGC7901/5-FU cells*

**Figure 1:**
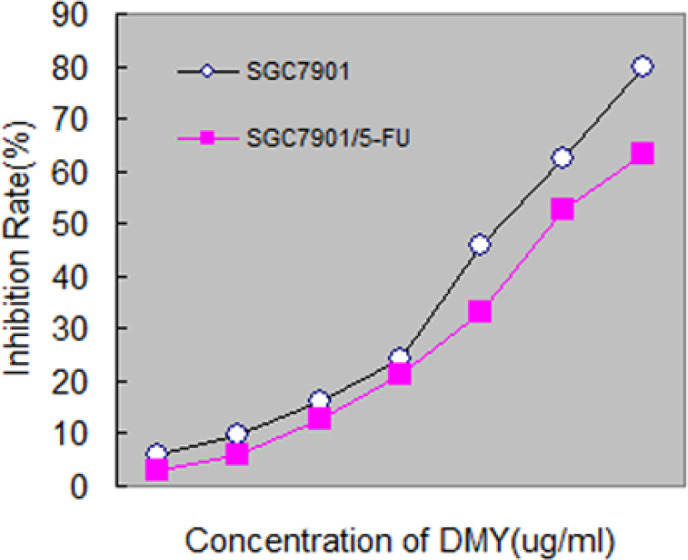
The Inhibition of DMY on SGC7901 and SGC7901/5-FU Cell Growth. DMY or RPMI1640 (control) were added to SGC7901 and SGC7901/5-FU cells for 48 h. MTT assay was performed to measure the cell proliferation in experiment and control groups, and the aforementioned fomular was used to calculate the inhibition rate (%). Each data point represents mean+SD of three wells

To examine the cytotoxicity of DMY against SGC7901 and SGC7901/5-FU cells, we used the MTT assay to investigate the growth of the cells exposed to various concentrations of DMY. Results showed DMY inhibited the proliferation of SGC7901 and SGC7901/5-FU cells in a dose-dependent manner in vitro, and the value of IC50 is 13.64±1.15 µg/mL, 20.69±1.82 µg/mL respectively (Figure 1). As shown in Fig 1, the concentrations below 2.5 μg/mL of DMY showed weakly cytotoxicity towards the SGC7901/5-FU cells (inhibition rate<15%). Thus, to evaluate the potent reversal effect of DMY against SGC7901/5-FU cells, The lower toxic concentrations of 1.25 μg/mL and 2.5 μg/mL DMY were selected.

*The MDR of SGC7901/5-FU cells to 5-fluorouracil*

 The 5-fluorouracil IC_50_ in SGC7901/5-FU and SGC7901 cells for 48 h were 54.37±11.04 µg/mL and 1723±132.25 µg/mL respectively. This means that SGC7901/5-FU was 31.7-fold resistance to the treatment of 5-FU in comparison with parental SGC7901 cells.

*DMY treatment sensitized SGC7901/5-FU cells to cytotoxicity of 5-FU*

To study whether DMY can enhance the cytotoxicity of 5-FU against SGC7901/5-FU cells, we combined 5-fluorouracil treatment with DMY at a nontoxic concentration of 1.25 μg/mL, or minimal effective concentration of 2.5 μg/mL. Table 1 shows the effect of DMY to enhance the sensitivity of SGC7901/5-FU cells to 5-fluorouracil. After the treatment with 5-fluorouracil at concentrations from 31.25 μg/mL to 2000 μg/mL and DMY at 1.25 μg/ml or 2.5 μg/ml in SGC7901/5-FU cells, the 5-FU IC50 values were decreased to 999.93±74.76 μg/mL and 530.24±48.04 μg/mL, respectively. The reversal effect of DMY on the sensitivity to 5-FU was evaluated with the reversal fold (RF), while RF>1 indicates the drug sensitivity was enhanced, RF=1 suggests no effect of the drug on the sensitivity, and RF<1 indicates the drug sensitivity was decreased (Brooks et al., 2003). The RF of DMY at concentrations of 1.25 μg/mL and 2.5 μg/mL were 1.72 and 3.25, respectively. These results indicated that DMY significantly increased the drug sensitivity of SGC7901/5-FU cells against 5-fluorouracil and reversed drug resistance.

*DMY enhanced the 5-fluorouracil-induced apoptosis*

**Figure 2:**
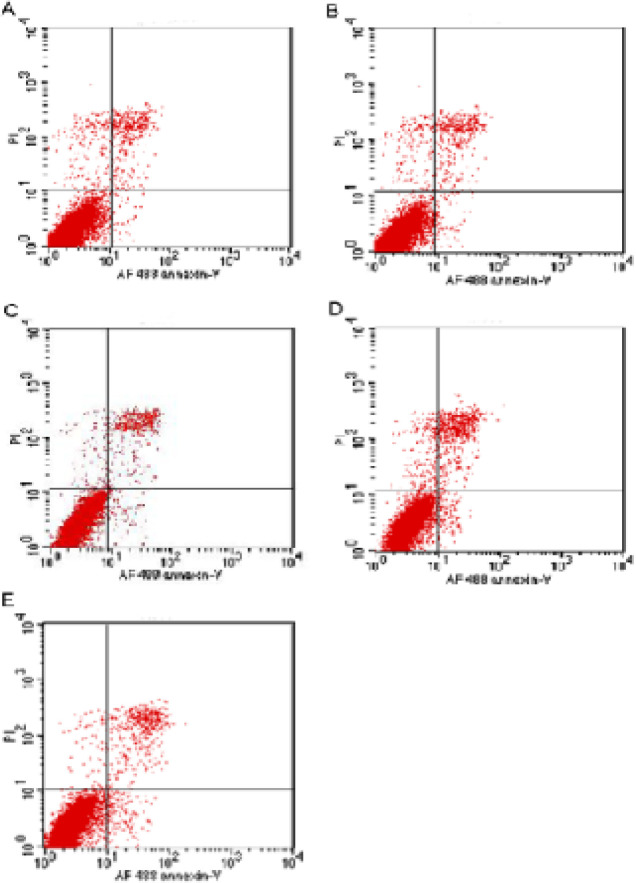
Effect of DMY on 5-Fluorouracil Induced Apoptosis by Flow Cytometry. (A) control; (B) DMY (1.25 µg/mL); (C) 5-FU (250 µg/mL); (D) DMY/5-FU (1.25 µg/mL/250 µg/mL); (E) DMY/5-FU (2.5 µg/mL /250 µg/mL)

5-fluorouracil-induced apoptosis of DMY against SGC7901/5-FU was analyzed by Annexin V staining and flow cytometry analysis after 24h treatment. As shown in Table 2 and Figure 2, after treatment with 5-fluorouracil (250 µg/mL) alone or in combination with DMY at 1.25 µg/mL or 2.5 µg/mL, the apoptosis rates were increased to 5.25%, 9.91% and 16.67%, respectively. The combination of DMY with 5-FU enhanced the apoptosis significantly in comparison with 5-FU alone (P<0.05), which contributed to the reversal effect of DMY against drug resistance in SGC7901/5-FU cells.

*Effects of DMY on the drug-resistant gene MDR1 expression*

**Figure 3:**
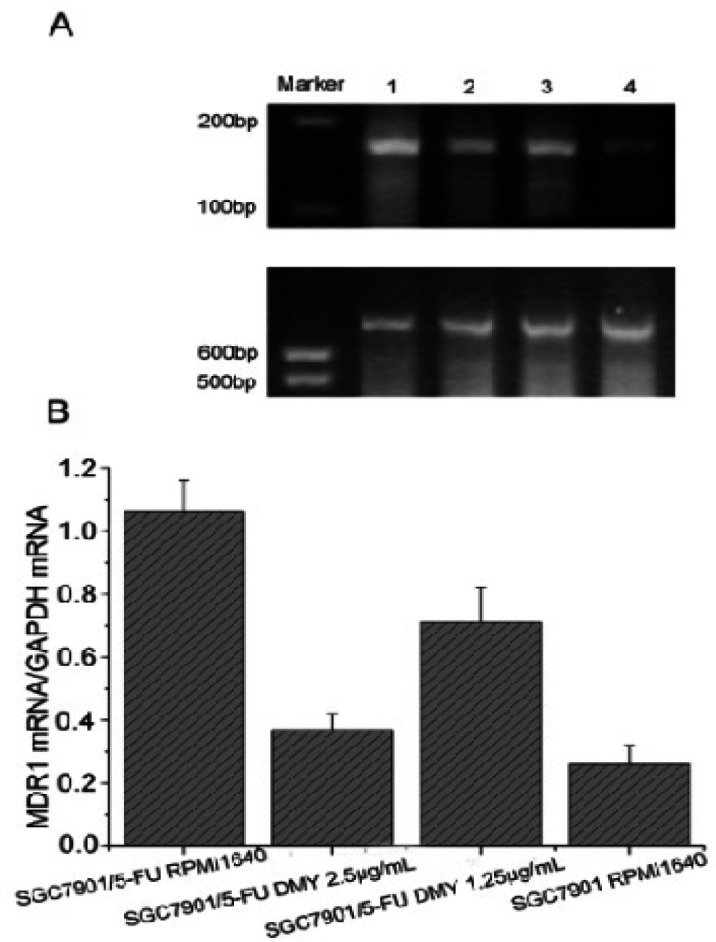
The MDR1 mRNA Expression in Experiment and Control Groups Cells and Inhibition of DMY on MDR1 mRNA Expression. (A) Picture of agarose gel electrophoresis. 1: SGC7901/5-FU RPMI1640; 2: SGC7901/5-FU DMY 2.5 μg/mL; 3: SGC7901/5-FU DMY 1.25 μg/mL; 4: SGC7901 RPMI1640. (B) The density of MDR1 bands in the agarose gel image was normalized by that of GAPDH bands. The values from 3 experiments were averaged and presented as mean+SD

**Figure 4:**
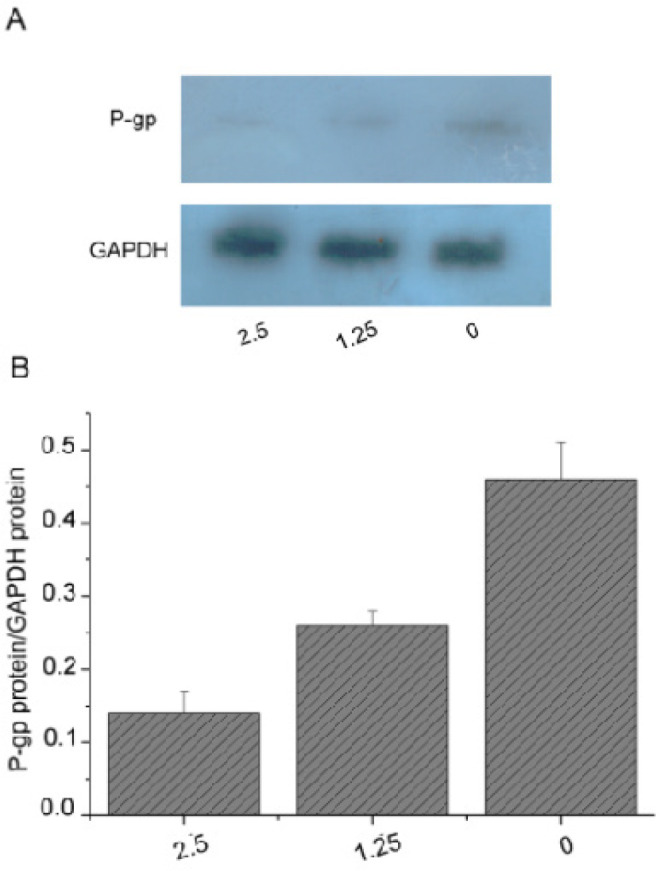
Inhibition of DMY on the Expression of MDR1/P-gp Protein in SGC7901/5-FU Cells. DMY or RPMI 1640 (control) were added to SGC7901/5-FU cells for 48 h. Protein were extracted and purified and then Western blot was used to determine P-gp expression. (A) Picture of enhanced chemiluminescence. (B) The density of P-gp bands in the agarose gel image was normalized by that of GAPDH bands. The values from 3 experiments were averaged and presented as mean+SD

To investigate potential mechanism of the enhancement of DMY on the cytotoxicity of 5-FU, multi-drug resistance 1 (MDR1) gene and protein expression were examined in the treatment of DMY. The mRNA level of MDR1 genes in SGC7901/5-FU cells was significantly higher than SGC7901 cells using RT-PCR (P<0.05) (Figure 3). After treatment with DMY (1.25 μg/mL or 2.5 μg/mL), the MDR1 expression level decreased significantly in comparison with the control group (P<0.05) (Figure 3). Western blotting showed a dose-dependent decrease of MDR1 (also known as P-gp) protein expression in SGC7901/5-FU cells in the treatment of DMY (P<0.05) (Figure 4). Taken together, these findings indicated that DMY may downregulate the mRNA and protein expression levels of MDR1, and attenuate drug resistant effect in SGC7901/5-FU cells. 

## Discussion

Globally, stomach neoplasms is one of the most frequent malignant tumors and the second major cause of death in diseases related to cancer (Torre et al., 2012). In general, surgery is the only curative therapeutic strategy for stomach neoplasms, but surgery is not suitable for all patients, while roughly half have inoperable tumors (Wang et al., 2019). For these patients, systemic chemotherapy are the primary treatment to pronlong survival (Okita et al., 2011). Nevertheless, chemotherapy has limited success because of multidrug resistance (MDR), which is a phenomenon tumor cells have the intrinsic or obtained resistance to various of anticancer drugs, thus the drugs failure to develop the anticancer effect. While increasing drug concentration leads to decreased curative efficacy and serious toxicity (Galati and Brien, 2004). Therefore, it is urgent to identify effective reversal agents to inhibit MDR with low toxicity.

Recently numerous studies showed natural products has been widely used for cancer treatment and served as potential reversal agents. DMY belongs to the flavonoids and a major bioactive component in Ampelopsis grossedentata (Du et al., 2002). It has been reported that Ampelopsis significantly inhibited proliferation of human promyelocytic leukemia HL-60 cells, human chronic myelogenous leukemia K562 cells and other cancer cells in vitro (Guamán-Ortiz et al., 2017). Our previous studies demonstrated that DMY combined with mitomycin has inhibitory effect on proliferation of gastric carcinoma cells (Wu et al., 2002). In the present study, we have shown DMY reversed the 5-FU resistance of SGC7901/5-FU cells. MTT assay results clearly revealed that DMY significantly inhibited proliferation of SGC7901 and SGC7901/5-FU cells in vitro in a concentration-dependent manner. In contrast, the multi-drug resistant SGC7901/5-FU cells were shown to be significantly more resistant to the cytotoxicity of 5-FU than its parent SGC7901 cell. On the other hand, treatment with low concentrations of DMY remarkably enhanced the cytotoxicity of 5-FU in the SGC7901/5-FU cells. As a result, DMY treatment reduced IC50 value of 5-FU and exhibited a dose-dependent increase of the reversal fold of the 5-FU cytotoxicity. Analysis of apoptosis indicated that combining DMY with 5-FU significantly increased apoptosis ratios in SGC7901/5-FU cells in comparison with 5-FU alone, suggesting the existence of the synergistic anticancer effect of DMY with 5-FU and the reversal of MDR. Several mechanisms were involved in the occurrence of MDR in cancer cells, the major cause was chemotherapy agents translocated from intracellular to extracellular, leading to the concentration of the anticancer agents reduced markedly, the phenomenon might chiefly attribute to P-glycoprotein (P-gp) which cause the agents outflow from the resistant cells actively (Weisburg et al., 1999). P-gp, a glycoprotein with 170-kDa molecular weight encoded by the MDR1 gene and consists of 12 transmembrane domains. P-gp plays an important role to pump various chemotherapeutic agents out of MDR carcinoma cells, including the following drugs: taxanes, Vinca alkaloids, anthracyclines, and epipodophyllotoxins (Sarkadi et al., 2006; Alameh et al., 2019; Qiu et al., 2019; Liu et al., 2010; Lopez-Chavez et al., 2009). The overexpression of P-gp endows cancer cell with MDR, thus various approaches were performed to investigate the decreasement of the P-gp expression and the inhibition or modulation of the P-gp activity (Alameh et al., 2019; Qiu et al., 2019). Recently many reports focus on the application of natural product such as flavonols or flavonoids, results demonstrated they had the decreased effect in the gene expression of multidrug resistance gene-1 (MDR1), and inhibit the transport activity and the of P-gp as well as the P-gp mediated drug efflux, consequently increase the intracellular concentration and cytotoxicity of chemotherapeutic agents, thus effectively reverse the resistance of MDR carcinoma cells in vitro (Liu et al., 2010; Lopez-Chavez et al., 2009). In order to explore whether DMY has similar reversal effects against anticarcinoma cells, we measured MDR1 expression treated by DMY. RT-PCR results suggested that DMY downregulate *MDR1 mRNA* expression of SGC7901/5-FU cells compared with SGC7901 cells. Furthermore, MDR1 protein expression was decreased significantly in SGC7901/5-FU cells after the treatment of DMY, indicating DMY exerts a reversal effect against resistance of 5-FU involved in the decreasing the mRNA and protein expression of MDR1 and consequently suppressing the pump effect of P-gp.

In conclusion, the data present in the present study indicate that DMY significantly enhanced the effects on proliferation inhibitory of 5-FU in SGC7901/5-FU cells in vitro, and reversed its resistance to 5-FU. One of the possible mechanisms of DMY against MDR was related to the inhibition of expression of MDR1, suggesting DMY may be a novel and effective MDR reversal agent for gastric cancer chemotherapy.

**Table 1:** Potency of DMY in Enhancing Cytotoxicity of 5-FU in SGC7901/5-FU Cells

concentration of DMY (μg /mL)	IC_50 _value of 5-FU (μg /mL)	RF
0	1723±132.25	
1.25	999.93±74.76*	1.72
2.5	530.24±48.04*	3.25

IC_50_ values are presented as the means±SD from at least three independent experiments. * indicates significant difference from the control group, P<0.05. RF stands for reversal fold

**Table 2:** Apoptotic of SGC-7901/5-FU Cells Induced by Single Drug Administration of DMY or 5-FU and Combination Treatment

Group	Apoptotic rate (% mean+SD)
Control	1.68±0.29
DMY (1.25 µg/mL)	3.43±0.59
5-FU (250 µg/mL)	5.25±0.89^∆^
DMY/5-FU (1.25 µg/mL /250 µg/mL)	9.91±0.87^∆^*
DMY/5-FU (2.5 µg/mL /250 µg/mL)	16.67±0.96^∆^*

^∆^ *P*<0.05; ^∆^* P<0.01 vs control; *P<0.05 vs 5-FU alone
